# First-in-human clinical study of an embryonic stem cell product for urea cycle disorders

**DOI:** 10.1186/s13287-025-04162-3

**Published:** 2025-03-06

**Authors:** Akihiro Umezawa, Akinari Fukuda, Reiko Horikawa, Hajime Uchida, Shin Enosawa, Yoshie Oishi, Naoko Nakamura, Kengo Sasaki, Yusuke Yanagi, Seiichi Shimizu, Toshimasa Nakao, Tasuku Kodama, Seisuke Sakamoto, Itaru Hayakawa, Saeko Akiyama, Noriaki Saku, Shoko Miyata, Kenta Ite, Palaksha Kanive Javaregowda, Masashi Toyoda, Hidenori Nonaka, Kazuaki Nakamura, Yoshikazu Ito, Yasuyuki Fukuhara, Osamu Miyazaki, Shunsuke Nosaka, Kazuhiko Nakabayashi, Chizuko Haga, Takako Yoshioka, Akira Masuda, Takashi Ohkura, Mayu Yamazaki-Inoue, Masakazu Machida, Rie Abutani-Sakamoto, Shoko Miyajima, Hidenori Akutsu, Yoichi Matsubara, Takashi Igarashi, Mureo Kasahara

**Affiliations:** 1 National Center for Child Health and Development, Setagaya, Japan; 2 National Center for Child Health and Development Research Institute, Setagaya, Japan; 3 Department of Surgery, Tohoku University Graduate School of Medicine, Sendai, Japan; 4 Department of Advanced Pediatric Medicine (National Center for Child Health and Development), Tohoku University School of Medicine, Sendai, Japan; 5 SDM Research Institute for Biomedical Sciences, A Constituent Unit of Shri Dharmasthala Manjunatheshwara University, Dharwad, India

**Keywords:** Hepatocyte, Embryonic stem cells (ESCs), Urea cycle disorders (UCD), Liver transplantation, Neonatal-onset, Ammonia removal, Hyperammonemia, Cell-based therapy, Regenerative medicine

## Abstract

**Background:**

This study assesses the safety and efficacy of hepatocyte-like cell (HLC) infusion therapy derived from human embryonic stem cells as bridging therapy for neonatal-onset urea cycle disorders (UCD). The research includes both preclinical and clinical evaluations to determine the feasibility of HLC infusion as a therapeutic option for safer pediatric liver transplantation.

**Methods:**

Preclinical studies were conducted to validate the safety, biodistribution, and ammonia metabolism capabilities of HLCs using SCID mice models of UCD and extensive animal studies. In the clinical trial, five neonates with UCD received HLC infusions, intending to maintain metabolic stability and exceed a target weight of over 6 kg, which is considered necessary for safer liver transplantation.

**Results:**

Preclinical studies demonstrated that HLCs successfully engrafted in the liver without adverse migration or tumor formation and effectively elongated survival. Clinically, all five neonates exceeded the target weight of 6 kg while maintaining metabolic stability and successfully bridging to transplantation. Post-transplantation follow-up revealed stable growth, metabolic control, and no neurological complications.

**Conclusions:**

The combined preclinical and clinical findings support HLC infusion as a viable bridge therapy for neonates with UCD, providing metabolic support to achieve safer weight thresholds for transplantation. While promising, careful monitoring remains essential, particularly for potential complications such as thrombus formation.

****Trial Registration**:**

jRCT, jRCT1090220412. Registered on 27 February 2019, https://jrct.niph.go.jp/en-latest-detail/jRCT1090220412 (originally registered in JMACCT (JMA-IIA00412)).

**Supplementary Information:**

The online version contains supplementary material available at 10.1186/s13287-025-04162-3.

## Background

Inborn errors of metabolism in the liver account for around 10% of pediatric liver transplantation cases [1]. The urea cycle is a biochemical pathway in the liver that involves six enzymes and two transporters, collectively converting ammonia into urea. Genetic mutations can result in UCDs such as ornithine transcarbamylase deficiency (OTCD), carbamyl phosphate synthase I deficiency (CPS1D), and citrullinemia type 1, all of which share the common feature of hyperammonemia [2]. The symptoms of these disorders include vomiting, hyperpnea, seizures, coma, and neurological impairment, which can be life-threatening and irreversible. The recommended treatment includes dietary therapy to restrict protein intake, drug therapy to promote ammonia excretion, and hemodialysis in acute cases. Liver transplantation is an effective and curative therapy [3].

Due to the high risk of severe postoperative complications in neonates, liver transplantation should be postponed until the patient reaches a weight of at least 6 kg. During the weight-gain period, hyperammonemia attacks can occur despite treatment, which may result in irreversible neurological impairment. Therefore, “bridge therapy” is necessary to ensure the patient’s safety before liver transplantation can be performed. In neonates with metabolic liver diseases, hepatocyte transplantation has been used in addition to conventional treatment [3, 4]. This approach offers several advantages, such as less invasive surgery compared to liver transplantation, the potential to use cells from a single donor liver for multiple patients, and the feasibility of cryopreservation for off-the-shelf use.

In addition to utilizing somatic cells such as hepatocytes, pluripotent stem cells may be employed. The first clinical trial of human embryonic stem cells (ESCs) was conducted ten years ago [5–9], and since then, ESC-derived products have been tested for various medical conditions, including age-related macular degeneration, Stargardt disease, type I diabetes, spinal cord injury, severe heart failure, and Parkinson’s disease. Our clinical trial utilized ESC-derived hepatocyte-like cells (HLCs) through intraportal injection for congenital metabolic disorders [5].

## Methods

### Participants

The patients were selected based on the inclusion and exclusion criteria, as outlined in Supplemental Information 1. We targeted patients with neonatal-onset congenital urea cycle disorders for enrollment in the first-ever clinical trial of an ESC-derived therapeutic product. The trial protocol was duly approved by the institutional review board of National Center for Child Health and Development (Tokyo, JAPAN) under the reference number [C29007].

This study was conducted with prior approval from Pharmaceuticals and Medical Devices Agency (PMDA) as a first-in-human trial to evaluate the safety and efficacy of HLC infusion therapy in a cohort of five patients. This initial case number was agreed upon with PMDA to ensure close monitoring and in-depth analysis of the therapy’s effects under strictly controlled conditions.

### Informed consent

As the subjects of this clinical trial were neonates and infants, informed consent was obtained in accordance with ethical standards. Specifically, informed consent for participation in the trial was obtained after thoroughly explaining the study to the surrogate (parents or legal guardians).

### Manufacturing

We utilized the ESC cell line SEES2 [10] to produce a master cell bank adhering to the guidelines of Good Manufacturing Practices or Good Clinical Practices. This cell line has undergone ex-vivo exposure with mouse embryonic fibroblasts and is thus classified as a xenotransplantation product [11]. HLCs were prepared from the starting material and characterized (Supplemental Information 1).

### Pre-clinical study

The efficacy of HLCs was examined by SCID-OTCD mice established in our institute [12]. The mice were administered 1 × 10^8 cells/kg of HCLs or cryopreserved human hepatocytes (In Vitro Technologies, Baltimore, MD, USA) into the spleen since the intrasplenic injection is known as a route of administration to the liver in rodents [13]. The number of administered HLCs was adjusted based on the estimated differentiation efficiency, as approximately 60% of the HLC population is expected to differentiate into functional hepatocytes. Mice were euthanized by cervical dislocation while under anesthesia with 3% isoflurane. Safety was verified in neonatal pigs using the same protocol for allogeneic hepatocytes previously [14], except that the dose was decreased by half (5 × 10^7). All animal experiments were approved by the Laboratory Animal Ethics Committee of the National Center for Child Health and Development (IRB number: A2000-001) based on the Japanese Guideline for Animal Experiments of Ministry of Health Labor and Welfare. The work has been reported in line with the ARRIVE guidelines 2.0.

### Clinical procedure

For the clinical study, we thawed, washed, and re-suspended cryopreserved HLCs in saline with 5% human albumin at a density of 1 × 10^7 cells/mL (Supplemental Fig. 1A–C). A tube containing the appropriate volume of formulated HLCs at 2–6 ℃ was transported to an operating room. The cells were injected with a double-lumen catheter (18G, Medicut LCV-UK; Tyco Healthcare, NJ, USA) inserted into the left portal vein via the umbilical vein [4]. The proximal end of the catheter was used for assessing portal pressure, while the distal end was used for HLC infusion. A T-shaped stopcock was connected to the inlet at the distal end of the cannula, and two syringes were set in the stopcock (Supplemental Fig. 1D). The 10-ml syringe was used as an HLC reservoir, and the 5-ml syringe was used as an injection pump. A portion of the cell suspension was transferred in the 5-ml syringe and carefully transfused to avoid excessive elevation of portal pressure (Supplemental Fig. 1E).

**Fig. 1:**
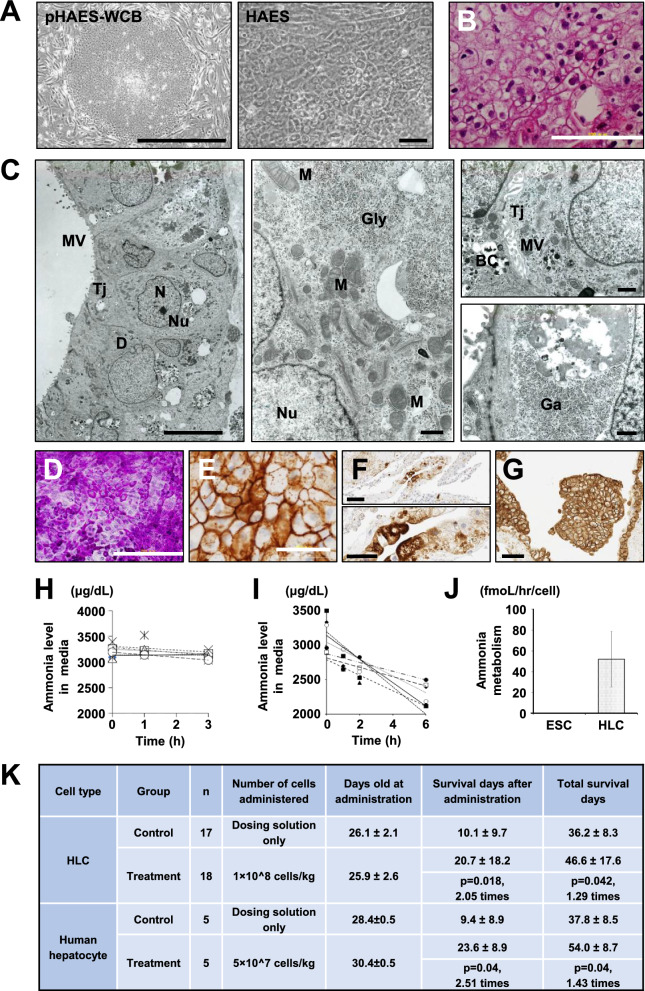
Characterization of HLCs. **A** Phase-contrast photomicrograph of undifferentiated ESCs (left: pHAES-WCB, Bar: 1.0 mm) and HLCs (right: HAES, Bar: 50 µm). **B** Histology of HLCs in iPGell. H.E. stain. Bar: 100 µm. **C** Ultrastructural analysis of HLCs. Left panel: HLCs showed polarity with microvilli on the apical membrane. HLCs had tight junctions and desmosomes between the cells. Bar: 10 µm. Middle panel: HLCs exhibited glycogen β-particles and had abundant mitochondria in the cytoplasm. Bar: 1.0 µm. Right upper panel: HLCs had microvilli at bile canaliculi and tight junctions between cells. Right lower panel: HLCs had large amounts of glycogen particles in the cytoplasm. N: nucleus, Nu: nucleus, Tj: tight junction, D: desmosome, MV: microvillus, G: golgi body, M: Mitochondria, BC: bile canaliculus. **D** Glycogen accumulation in HLCs. Periodic acid–Schiff (PAS) stain. Bar: 200 μm. **E** Immunohistochemistry of HLCs with an antibody to E-cadherin. Bar: 50 μm. **F** Immunohistochemistry of HLCs with an antibody to AFP. Bar (upper panel): 100 μm, Bar (lower panel): 50 μm. **G** Immunohistochemistry of HLCs with an antibody to cytokeratins (AE1/3). Bar: 100 μm. **H**, **I** Time-course analysis of ammonia concentration was measured in culture media of undifferentiated ESCs (H) and HLCs (I). **J** Ammonia metabolic activity of undifferentiated ESCs and HLCs was summarized. **K** Effect of human hepatocyte-like cell treatment on the survival of SCID-OTCD mice compared with human cryopreserved hepatocytes

A dose of 1 mL of HLCs at a concentration of 1 × 10^7 cells/mL was administered over 1–5 min, followed by a 2 to 10-min dosing pause. For the injection of HLCs at a dose of 5 × 10^7 cells/kg, the lateral segment of the liver was chosen instead of the entire liver to maintain liver function even if dysfunction occurred in a part of the left lobe of the liver. HLCs were infused in two doses, with 2.5 × 10^7 cells/kg administered in the first and second doses. The second dose was administered 2–3 days after the first dose, and before the start of the second dose, the portal pressure was confirmed to be within 1.25 times the value before the first dose, and the portal blood flow velocity was at least 0.75 times higher. Portal vein pressure was measured through one channel of the double-lumen catheter, and HLCs were slowly injected through the other channel. Portal blood flow was monitored using color Doppler ultrasonography. If portal venous blood flow velocity experienced a decrease of 50% or greater from the pre-administration value, the administration was temporarily suspended. The blood flow velocity was measured at 10-min intervals (± 3 min), with the recorded value in the case report form, and the administration was resumed when the decrease was 25% or less [15]. If the portal vein pressure increased by 50% or more from the pre-administration value, the administration was temporarily suspended, and administration was resumed when the increase was 25% or less.

To avoid systemic circulation of HLCs through an open ductus venosus, portal venography was performed to check for patency of the ductus venosus. If the ductus arantius was open, it was closed before injection. This protocol is based on our experience safely administering human allogeneic hepatocytes to neonates [4]. The first HLC infusion was performed under sedation and ventilator control, and the second and subsequent infusion was performed without ventilator control in the intensive care unit using an indwelling catheter.

### Immunosuppressive treatment

Tacrolimus and prednisolone were used as immunosuppressants. The dosage and administration of steroids and the targeted trough values of tacrolimus were based on previous studies [16–19].

## Results

### Characterization of HLCs

SEES2 ESCs were differentiated into HLCs (Supplemental Information 1). HLCs expressed high levels of AFP, a marker for hepatoblasts, and had the capability to metabolite ammonia. HLCs exhibited hexagonal structures with well-defined cell boundaries and cell nuclei with delicate chromatin and distinct nucleoli (Fig. 1A, B). Ultrastructural analysis revealed that HLCs exhibited bile canaliculi-like structures, typical junctional complexes, and microvilli (Fig. 1C). HLCs contained numerous glycogen granules in the cytoplasm and subcellular organelles, such as mitochondria, peroxisomes, lysosomes, and endoplasmic reticulum. HLCs contained glycogen in the cytoplasm by PAS stain (Fig. 1D). Immunocytochemistry revealed that HLCs were positive for E-cadherin, α-fetoprotein, and cytokeratins (AE1/3) (Fig. 1E–G). HLCs metabolized ammonia in vitro (Fig. 1H–J). The protocol achieves about 60% differentiation efficiency of ESCs into HLCs, as determined morphologically.

### Toxicology, tumorigenicity, and biodistribution

Non-clinical studies on safety were performed in an external GLP-compliant facility. A single dose of 5.5 × 10^7 cells/kg was injected via the portal vein into male and female nude rats, and toxicity was studied for 12 weeks after the injection (Supplemental Information 1). No changes suggestive of toxicity were observed. We then performed biodistribution studies by using DNA quantitative PCR, designed to amplify human Alu DNA sequences in nude rats (Supplemental Information 1) [20]. No injected cells were detected in all organs except the liver, implying that the injected cells reach the liver through the portal vein and do not migrate to the other organs. Additionally, HLCs had no tumorigenic potential at 16 weeks after a single dose of 1 × 10^7 cells was subcutaneously injected into nude mice whereas HeLa S3 cells formed tumors in all cases. Injection of undifferentiated ESCs at a ratio of 1%, 10%, or 100% to the number of HLCs all formed teratomas at the subcutaneous sites. Likewise, HLCs showed no tumorigenic potential at 12 weeks after injection.

### Implantation of HLCs into immunodeficient ornithine transcarbamylase-deficient mice (SCID-OTCD mice)

To assess efficacy, HLCs were injected into the spleen of 20–30 days old SCID-OTCD mice at a concentration of 1 × 10^8 cells/kg (2 × 10^4 cells/μL) (Fig. 1K) because hepatocytes injected into the spleen are expected to migrate to the liver via a splenic vein [13]. Human hepatocytes were also administered to SCID-OTCD mice for comparison. Sham control groups were injected with the same volume of solution for suspension. The dose of HLCs was determined as 5 × 10^7 cells/kg, based on the provisional dose for hepatocyte transplantation [14]. Both overall survival and post-dose survival were significantly prolonged in the injection group compared to the control group. HLC administration increased the mean number of days of overall survival and post-administration survival by 1.29-fold and 2.05-fold, respectively. In contrast, human hepatocyte injection increased the mean number of days of overall survival by 1.43-fold and the mean number of days of post-administration survival by 2.51-fold.

In the mice experiments, variability in survival days and ammonia levels was observed among the treatment group. This variability likely reflects biological differences in baseline metabolic function and responses to HLC therapy, engraftment efficiency, functional integration, and host immune responses. While this variability is a limitation, it offers valuable insights into the challenges of applying such therapies to heterogeneous populations. SCID-OTCD mice do not exhibit liver failure; the engraftment of hepatocytes and HLCs is expected to be limited. Since the liver function in this model is normal, it is challenging for transplanted hepatocytes and HLCs to engraft long-term, and this is considered one of the main reasons for the observed loss of function. While transplanted HLCs and hepatocytes perform a temporary functional role shortly after transplantation, maintaining long-term engraftment and function proves difficult. Factors such as apoptosis, immune rejection, and ischemia may significantly influence this process.

### Clinical course

**Fig. 2:**
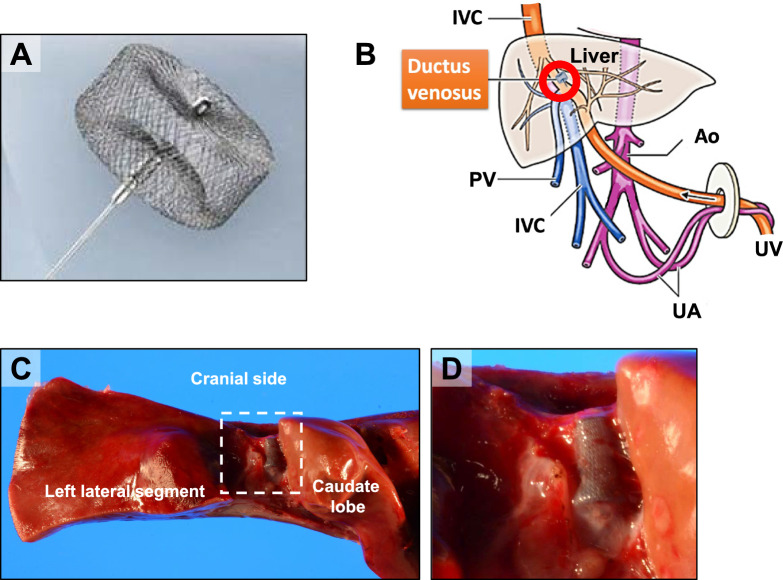
Embolization using a vascular plug under IVR. **A** Macroscopic view of AmplatzerTM Vascular plug used for embolization. To avoid systemic circulation of HLCs, an open ductus venosus is closed before injection. **B** Embolization of ductus venosus. Ductus venosus is closed 15–20 days after birth [26]. The ductus venosus was patent in Cases 1 and 2 and thus was embolized before the transplantation. **C** AmplatzerTM Vascular plug in the explanted liver. **D** High-power view of AmplatzerTM Vascular plug (C, dotted rectangle)

We used the umbilical route and investigated the ductus venosus opening to avoid extrahepatic migration. When the ductus venosus was open, we blocked the route with an embolization material called a vascular plug prior to the infusion (Fig. 2). This procedure is minimally invasive and can thus be performed on newborns without any problems. Indeed, the Case 2 patient received the procedure at 2 days old. Furthermore, the infusion route was done via the umbilical portion, which serves as the left portal vein entrance. Even if cells embolize, ischemia is confined to the left lateral segment, blood flow to the portal vein is assured, and ischemia of the entire liver is avoided.

**Fig. 3:**
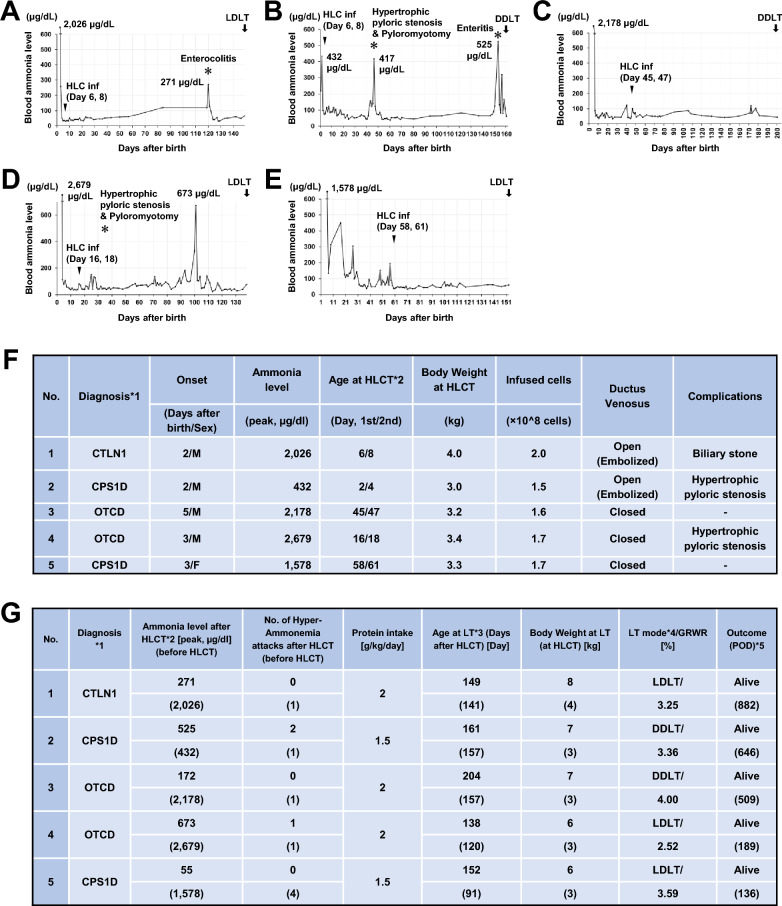
Clinical courses of HLC treatment. **A**–**E** Profiles of blood ammonia levels of the patients with urea cycle disorder from the onset of the disease to liver transplantation. Arrowheads indicate the time of HLC infusion (HLC inf), and the numbers in parentheses are the ages and days of transplantation. Arrows indicate the time of liver transplantation. LDLT: living donor liver transplantation (**A**, **D**, **E**), DDLT: deceased donor liver transplantation (**B**, **C**). Values at marked hyperammonemia seizures are shown in the graph. Asterisks indicate major clinical episodes; enterocolitis (**A**), enteritis (**B**), hypertrophic pyloric stenosis, and pyloromyotomy (**B**, **D**). **F** A list of human hepatocyte-like cell treatments. *1 CTLN1: citrullinemia type 1, CPS1D: carbamoyl phosphate synthetase 1 deficiency, OTCD: ornithine transcarbamylase deficiency, *2 HLCT: hepatocyte-like cell treatment. **G** Clinical course after hepatocyte-like cell treatment and liver transplantation. *1 CTLN1: citrullinemia type 1, CPS1D: carbamoyl phosphate synthetase 1 deficiency, OTCD: ornithine transcarbamylase deficiency, *2 HLCT: hepatocyte-like cell treatment, *3 LT: liver transplantation, *4 LDLT: living donor liver transplantation, DDLT: deceased donor liver transplantation, GRWR: graft-recipient weight ratio, *5 POD: postoperative day

Five patients with neonatal-onset urea cycle disorders underwent HLC infusion (Fig. 3, Supplemental Information 1). In all cases, a small skin incision of approximately 2 cm was made above the umbilicus under general anesthesia. The umbilical vein was then accessed and cannulated into the portal vein without the need for laparotomy. Case 1 had a seizure on day 2 and was started on hemodiafiltration because of an ammonia level of 2,026 µg/dL. The high urinary orotate and markedly elevated uracil and citrulline levels led us to suspect citrullinemia type 1, and the genetic mutation was confirmed at 6 days old. HLCs were infused twice at 6 and 8 days old. Initial ammonia levels of 2,026 µg/dL stabilized below 150 µg/dL after infusion, while protein intake increased from 0.9 to 2.0 g/kg/day by 43 days old. The patient exhibited no significant liver function abnormalities and achieved adequate growth for successful liver transplantation. Case 2 was a boy whose prenatal diagnosis by chorionic villus biopsy showed the same genetic mutation as that of his sister because his sibling had received a living donor liver transplantation for CPS1D. The boy was diagnosed with CPS1D by genetic testing of his blood after birth and was infused with HLCs at 2 and 4 days old. Initially measured at 432 µg/dL, ammonia levels spiked transiently to 417 µg/dL post-surgery, while protein intake improved from 0.25 to 1.5 g/kg/day. Case 3 had seizures and hyperammonemia at 5 days old, and continuous hemodiafiltration and drug therapy were started. A genetic test confirmed the diagnosis of OTCD at 12 days old. The patient was weaned from hemodiafiltration dialysis and continued on pharmacological treatment. HLCs were infused at 45 and 47 days old. Ammonia levels were maintained at 40 µg/dL, while protein intake rose from 1.0 to 2.0 g/kg/day by 48 days old. Case 4 was found to have poor vitality, respiratory failure, and hyperammonemia and was started on continuous hemodiafiltration in addition to drug therapy. At 3 days old, the patient was on a ventilator for respiratory failure and hyperammonemia, and continuous hemodiafiltration was started in addition to drug therapy. At 6 days old, genetic testing revealed OTCD, and HLCs were infused at 16 and 18 days old. The cell infusion resulted in ammonia level reductions and stable protein intake without restrictions. The patient exhibited minor growth delays but no significant developmental issues. Case 5 was placed on a ventilator at 3 days old due to decreased feeding and hyperpnea. Amino acid analysis revealed high levels of glutamine, glutamic acid, alanine and low citrulline levels. She was suspected of having CPS1D and was treated with medication and continuous hemodiafiltration. Initial ammonia levels of 1,578 µg/dL stabilized, and protein intake increased from 1.0 to 1.5 g/kg/day by 70 days old. MRI revealed cerebral atrophy and spiny EEG waves associated with prior hyperammonemia. After four episodes of recurrent hyperammonemia following weaning from dialysis, progressive neurological impairment was suspected. As a genetic mutation in CPS1 was confirmed, HLCs were infused at 58 and 61 days old.

**Fig. 4:**
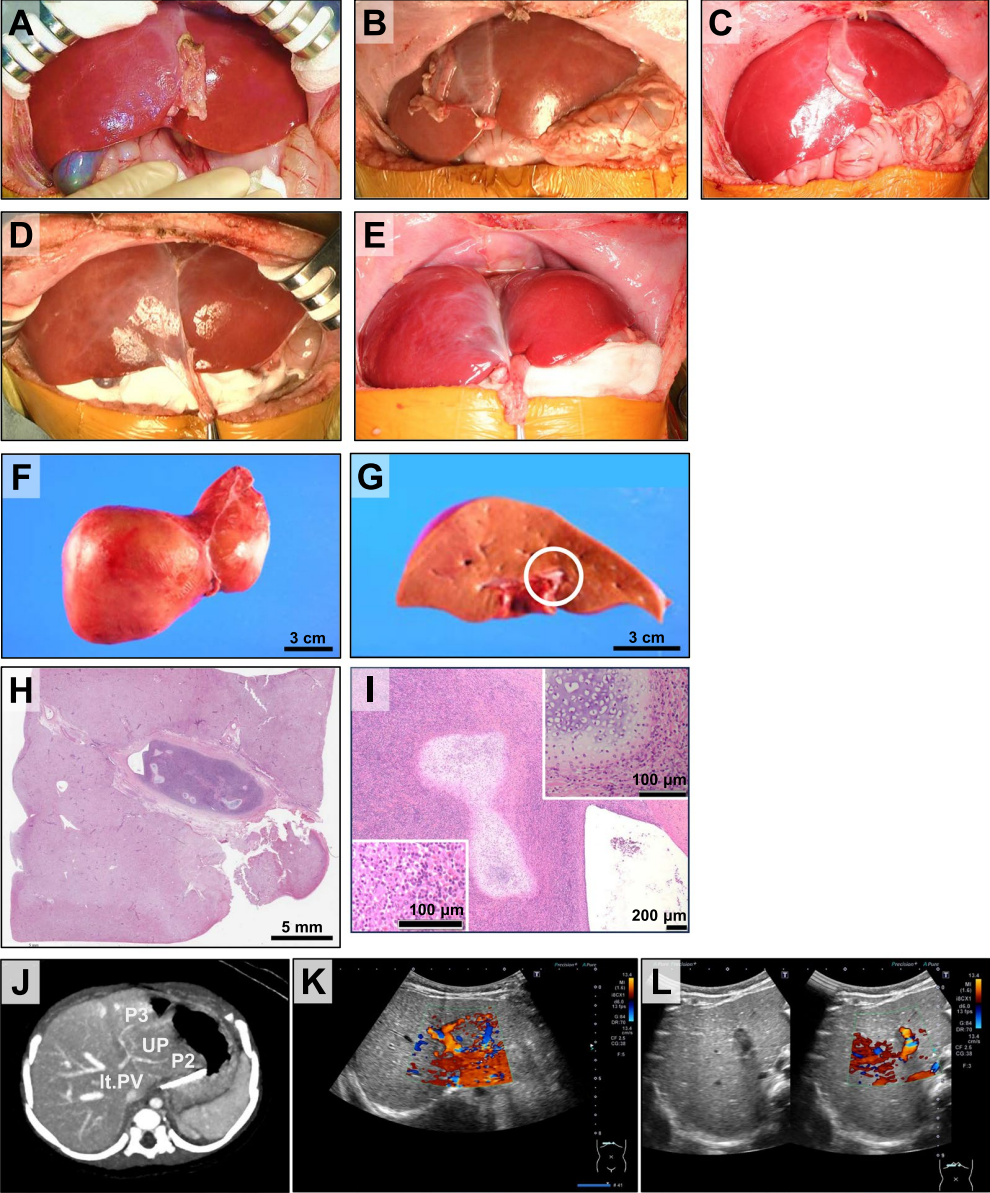
Macroscopic and microscopic view of the explanted liver in Case 4, OTCD. **A**–**E** Macroscopic anterior view of livers. The patients’ livers that received ESC-derived hepatocytes showed a smooth and normal surface in all cases upon macroscopic examination, despite a functionally disabled urea cycle, just before they were resected for transplantation. No discoloration or ischemic changes were detected; no tumors were formed. **A** Case 1: CTLN1, **B** Case 2: CPS1D, **C** Case 3: OTCD, **D** Case 4: OTCD, **E** Case 5: CPS1D. **F** Macroscopic view of the superior surface of the explanted native liver in Case 4. **G** A white thrombus (white circle) in the umbilical portion of the portal vein upon examination of the cut surface of the explanted native liver in Case 4. **H** Low-magnification histopathological analysis of the thrombus in Case 4. **I** Chondrogenesis observed within the embolus. Upper right panel: chondrogenesis. Lower left panel: inflammatory cell infiltration in Case 4. **J** Contrast-enhanced CT scan in Case 4. Lt. PV: left portal vein, UP: umbilical portion. **K** Evaluation of portal vein flow 2 months after the hepatocyte transplantation in Case 4. **L** Evaluation of portal vein flow at three months post-hepatocyte transplantation, or one-month pre-liver transplantation in Case 4

All the patients exceeded the target body weight of 6 kg at a median age of 152 (138–204) days, and liver transplantation from 3 living and 2 brain-dead donors was performed (Fig. 3G). At the time of liver transplantation, there was no color change, atrophy, or hypertrophy on the liver surface of the left lobe where HLCs were infused (Fig. 4A–E). Histopathologic examination of the explanted liver revealed thrombi/emboli in two cases (P3 and P4). In the fourth case, an embolus was found in the central portal vein accompanied by chondrogenesis (Fig. 4F–I). The embolization exhibited an inflammatory reaction with lymphocytic infiltration, suggestive of rejection. Chondrogenesis was also observed in the peripheral portal vein. No evidence of differentiated cells other than hepatocytes and chondrocytes was detected, and no clear teratoma formation was observed. Additionally, no ischemic changes were noted in any of the cases.

In Case 4, an embolism was observed in the portal vein, prompting a reevaluation of the portal vein blood flow between the hepatocyte transplantation and liver transplantation using contrast-enhanced CT scans and ultrasound. The contrast-enhanced CT scan at three months post-hepatocyte transplantation showed luminal narrowing indicative of an embolism (Fig. 4J). Furthermore, ultrasound imaging at two months post-hepatocyte transplantation did not reveal a clear embolism (Fig. 4K). However, at three months post-hepatocyte transplantation or one month before liver transplantation, luminal narrowing was observed (Fig. 4L).

In this study, examination of the explanted liver tissue revealed no detectable HLCs, suggesting that their engraftment duration was limited. This finding supports the hypothesis that HLCs serve a temporary functional role as a bridge to transplantation, and the HLCs did not persist long-term nor undergo significant proliferation. These results are consistent with the fact that the target patients often did not exhibit liver dysfunction, which may have limited the engraftment and long-term retention of HLCs. Furthermore, lymphocytic infiltration observed in some cases suggests that immune-mediated rejection may have contributed to the disappearance of HLCs. To address these limitations, future studies will incorporate molecular analyses, such as monitoring cell-free DNA derived from transplanted cells in plasma and performing enzyme assays specific to the transplanted cells. These approaches are expected to provide more sensitive methods for detecting functional HLCs over time.

## Discussion

Pediatric living donor liver transplantation has been utilized for patients with conditions such as biliary atresia, fulminant hepatic failure, and congenital metabolic liver diseases [21]. For patients with congenital metabolic liver diseases, hepatocyte transplantation is an effective bridge therapy [3, 22, 23]. Although hepatocyte transplantation has been performed in about 100 cases worldwide for various diseases, metabolic liver disease has shown the most promising results. Current clinical targets for hepatocyte transplantation include alpha 1-antitrypsin deficiency, Crigler-Najjar syndrome type I, and ornithine transcarbamylase deficiency (OTCD). Our team performed hepatocyte transplantation in two neonates with OTCD and carbamoyl phosphate synthetase 1 deficiency (CPS1D), both suffering from hyperammonemia, as a bridge to liver transplantation [24]. Although a relatively small number of cells were infused (1–2% of the total hepatocytes in the recipient’s liver), this therapy stabilized the patients’ clinical conditions.

Hepatocyte transplantation can pose challenges due to the shortage of donors, and the difficulty in securing a sufficient number of hepatocytes with consistent quality. To address this issue, this study utilized human ESC-derived HLCs, and in 3 out of 5 cases, stable management without hyperammonemia attacks requiring hemodiafiltration was achieved. One patient had no episodes of hyperammonemia after HLC infusion despite requiring hemodiafiltration four times before the procedure. All patients avoided fatal hyperammonemia and irreversible neurological impairment after HLC infusion and achieved weight gain of up to 6 kg, which is relatively safe for liver transplantation. HLC therapy can serve as a bridge therapy to liver transplantation, providing an alternative treatment option. To ensure efficacy, it is assumed that cell engraftment plays a critical role. Monitoring the transplantation and rejection of administered hepatocytes may eventually include cell-free liquid biopsy [25]. Advances in liquid biopsy could offer new useful non-invasive markers for regenerative medical products. By using blood samples, it may be possible to sequentially monitor hepatocyte engraftment and rejection through DNA polymorphisms derived from donor hepatocytes present at specific time points [25].

Of concern is the presence of an intraportal embolus in the removed liver of the fourth patient. We previously reported an intraportal embolus associated with hepatic ischemia in a hepatocyte transplant. Hepatocyte transplantation has been reported to decrease portal vein blood flow, but there are no reports specifically on thrombosis [26, 27]. In the present case, no evidence of hepatic ischemia was observed. During the follow-up echocardiogram, only findings suggestive of stenosis at the central portal vein were observed. Therefore, the thrombus may occur with cartilage formation between cell transplantation and liver transplantation [4]. There is a study using primates that hepatic ischemia increases hepatocyte engraftment [28]. However, considering the risks associated with ischemia, stringent process control in manufacturing processes is crucial at this stage. This necessitates sufficient pre-shipment quality testing as a quality control measure to verify gene expression at all manufacturing steps and to determine whether the expression of differentiation markers other than those specific to hepatocytes is elevated. The absence of teratoma formation aligns with the lack of expression of undifferentiated markers of pluripotent stem cells. Additionally, the safety of HLCs, including their tumorigenicity, was demonstrated in a preclinical study using nude rats in a GLP-compliant facility.

Acknowledging the importance of long-term safety, particularly regarding tumorigenicity, we initiated a follow-up study to systematically assess the tumorigenic potential of ESC-derived HLCs. This follow-up study is designed to monitor patients over an extended period to detect any delayed adverse events, including tumor formation. The findings from this ongoing research will contribute to a deeper understanding of the long-term safety profile of ESC-derived therapies and will be invaluable for informing future clinical applications and regulatory guidance.

## Conclusions

This study evaluates the safety and efficacy of ESC-derived HLCs in patients with urea cycle disorders. The livers of five patients infused with HLCs showed no problematic pathological findings of abnormal proliferation or teratoma formation. Liver transplantation is an effective curative therapy for hyperammonemia due to urea cycle disorders. When liver transplantation is difficult due to the recipient’s low weight or complications, cell transplantation provides a bridge to liver transplantation, and this clinical trial showed that ESC-derived HLCs are as effective as hepatocytes derived from surplus liver. The ultimate therapeutic goal is to achieve safe bridging without irreversible neurological impairment. The ammonia levels in all patients remained stable for a median duration of 1 year and 5 months (136–882 days), and the clinical trial is ongoing with continuous follow-up, including monitoring for neurological damage.

## Supplementary Information

Additional file1

Additional file2

Additional file3

Additional file4

Additional file5

Additional file6

Additional file7

Additional file8

## Data Availability

The datasets used and/or analyzed during the current study are available from the corresponding author on reasonable request.
