# Supplementary material for: First-in-human clinical study of an embryonic stem cell product for urea cycle disorders
Source: Stem Cell Res Ther. 2025 Mar 6;16:120. doi: 10.1186/s13287-025-04162-3 (PMC11887382; doi:10.1186/s13287-025-04162-3)
Supplement: Supplementary file 1 — Additional file1 [file 13287_2025_4162_MOESM1_ESM.pdf]

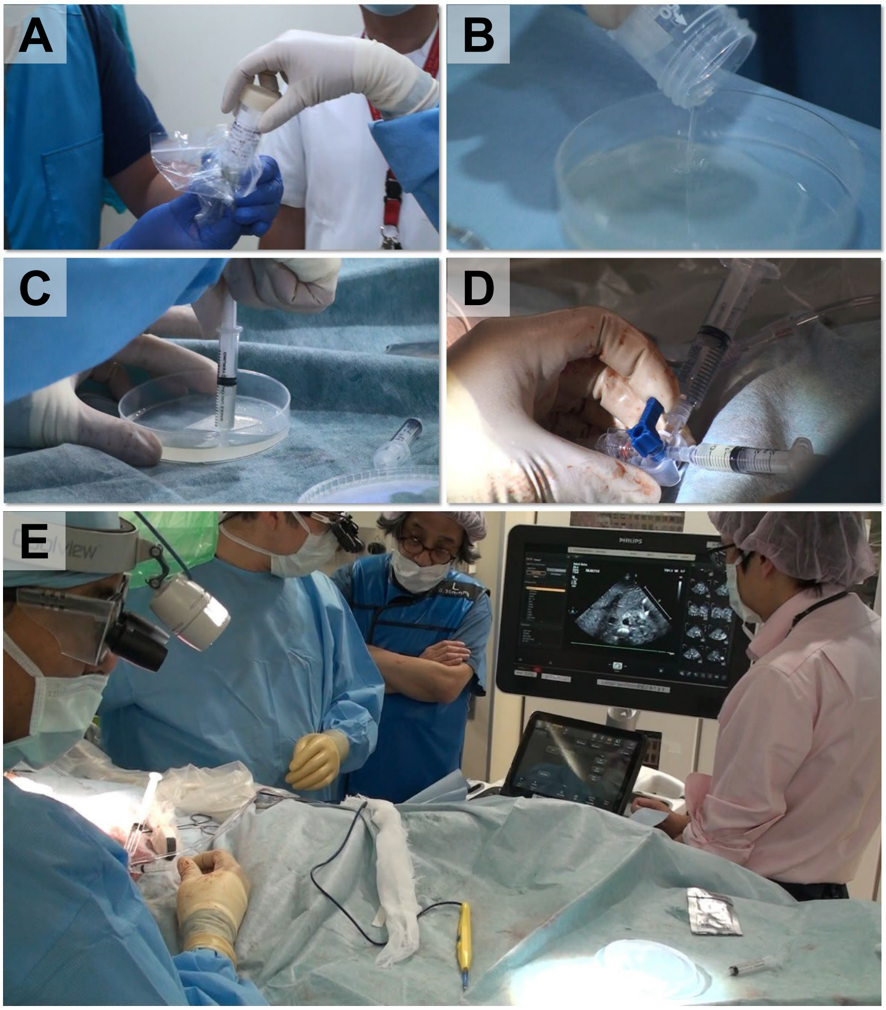

Supplemental Figure 1. Transplantation of ESC-derived hepatocytes via umbilical vein

(A) A tube containing the appropriate volume of formulated HLCs at 2-6℃ was transported to an operating room. **(B)** Hepatocyte-like cells (HLCs) in a cellular suspension are transferred from a 50-milliliter tube to a 10-centimeter dish. **(C)** The cell suspension was transferred into a 10-milliliter injection syringe. **(D)** A T-shaped stopcock was connected to the inlet at the distal end of the cannula, and two syringes were inserted into the stopcock. The 10-milliliter syringe served as an HLC reservoir, while the 5-milliliter syringe functioned as the injection pump. **(E)** HLCs were carefully transfused to avoid excessive elevation of portal pressure.
