# Supplementary material for: First-in-human clinical study of an embryonic stem cell product for urea cycle disorders
Source: Stem Cell Res Ther. 2025 Mar 6;16:120. doi: 10.1186/s13287-025-04162-3 (PMC11887382; doi:10.1186/s13287-025-04162-3)
Supplement: Supplementary file 2 — Additional file2 [file 13287_2025_4162_MOESM2_ESM.pdf]

Supplemental Information 1.
Characterization and Quality Control of Human Embryonic Stem Cell-Derived Product for Therapeutic Application

Table 1 Test for the cell bank

	Test	MCB	WCB	CAL
Specifications	Morphology	✓	✓	-
	Teratoma formation	✓	-	-
	DNA fingerprinting (Short tandem repeat)	✓	-	-
	Hepatocyte markers, stem cell markers, ammonia metabolism, secretion of non-purpose bioactive substances	✓	✓	✓
Purity	Sterility	✓	-	✓
	Mycoplasma	✓[a]	✓[a]	✓[b]
	Human virus	✓	-	✓
	Mouse virus	✓	-	✓
Characterization	Embryoid body formation (*in* vitro)	✓	-	-
	Expression of ESC specific markers (histological)	✓	-	-
	Expression of ESC specific markers (mRNA)	✓	-	-
	Karyotypic analysis	✓	-	-
	Comparative genomic hybridization analysis	✓	-	-
	Gene chip analysis	✓	-	-
	Exome analysis	✓	-	-

a) Japanese Pharmacopoeia (JP) 17th Ed. Method C
b) JP 17th Ed. Method A and B https://www.mhlw.go.jp/file/06-Seisakujouhou-11120000-Iyakushokuhinkyoku/JP17_REV_1.pdf

Table 2 Characterization of pHAES Cell Bank

Test		Method	pHAES MCB
Sterility		EP/USP - direct inoculation and membrane filtration methods	Negative
Mycoplasma		JP Reference information - Culture method, DNA staining method, and Nucleic acid amplification technique (Method B & C).	Negative
Human virus		Detection by qPCR: Hepatitis B virus, Hepatitis C virus, Human immunodeficiency virus type 1 and type 2, Human T-cell lymphotropic virus type 1 and type 2, Human parvovirus B19	Negative
Mouse virus	Retroviruses	Co-cultivation with Retrovirus-sensitive *Mus Dunni* and PG-4 cells	Negative
	Infectious diseases	Detection of viral reverse transcriptase by PCR	Negative
	Electron microscopy	Cellular morphology and detection of viral particles	Negative
	In vitro detection	Incubation with MRC-5 cells, Vero cells and NIH-3T3 cells, haemadsorption and haemagglutination	Negative
	In vivo detection	Inoculation into suckling and adult mice, guinea pigs and embryonated hen eggs (allantoic and yolk sac routes)	Negative
	Antibody production (mouse)	Antibody titers on inoculated mice for 17 viruses (Sendai Virus, Pneumonia Virus of Mice, Mouse Hepatitis Virus, Minute Virus of Mice, Mouse Parvovirus, Mouse Poliovirus (GDVII), Reovirus Type3, Epizootic Diarrhea of Infant Mice, Mouse Pneumonitis Virus (K virus), Ectromelia, Polyoma Virus, Mouse Adenovirus, Lymphocytic Choriomeningitis Virus (LCMV), Mouse Cytomegalovirus, Mouse Thymic Virus, Hantaan Virus, Prospect Hill Virus)	Negative

Generation of pHAES Master Cell Bank

The ESC line was used in compliance with "the Guidelines for Derivation and Distribution of Human Embryonic Stem Cells (Notification of the Ministry of Education, Culture, Sports, Science, and Technology in Japan (MEXT), No. 156 of August 21, 2009; Notification of MEXT, No. 86 of May 20, 2010) and "the Guidelines for Utilization of Human Embryonic Stem Cells (Notification of MEXT, No. 157 of August 21, 2009; Notification of MEXT, No. 87 of May 20, 2010)" [1–3]. SEES-2 seed stock was thawed and expanded through four serial passages on irradiation-inactivated MEFs. The clinical pHAES master cell bank (pHAES-MCB) was cryopreserved and confirmed to have a normal female (46, XX) karyotype with blood type O and to be free of bacterial and mycoplasmal contaminants as well as human, bovine, porcine and murine viruses [2]. Whole genome analysis showed no changes or mutations in genes associated with the urea cycle, including OTC, CPS1, ASS, ASL, and ARG1, and in 614 cancer-related genes [4,5].

Table 3 Characterization of MEF Master Cell Bank

Test			Method	Specification
Safety and purity	Sterility		EP/USP - direct inoculation and membrane filtration methods	Negative
	Mycoplasma		JP Reference information - Nucleic acid amplification technique (Method C).	Negative
	Mouse virus	Retrovirus	Co-cultivation with Retrovirus-sensitive *Mus Dunni* and PG-4 cells	Negative
		Retrovirus	PCR-based viral reverse transcriptase detection	Negative
		Retrovirus	Ultrastructural electron microscopy of cellular morphology and detection of viral particles	Negative
		In vitro detection	Incubation with MRC-5 cells, Vero cells and NIH-3T3 cells, haemadsorption and haemagglutination	Negative
		In vivo detection	Inoculation into suckling and adult mice, guinea pigs and embryonated hen eggs (allantoic and yolk sac routes)	Negative
		Antibody Production (mouse)	Antibody titers on inoculated mice for 17 viruses (Sendai Virus, Pneumonia Virus of Mice, Mouse Hepatitis Virus, Minute Virus of Mice, Mouse Parvovirus, Mouse Poliovirus (GDVII), Reovirus Type3, Epizootic Diarrhea of Infant Mice, Mouse Pneumonitis Virus (K virus), Ectromelia, Polyoma Virus, Mouse Adenovirus, Lymphocytic Choriomeningitis Virus (LCMV), Mouse Cytomegalovirus, Mouse Thymic Virus, Hantaan Virus, Prospect Hill Virus)	Negative
Performance qualification	Phase contrast microscope morphological observation		Ability of MEFs to support growth and attributes of human ESCs (pHAES) in culture	Pass

Mouse embryo fibroblasts (MEFs) master cell bank (MCB) for pHAES cells

MEFs derived from 12.5- to 13.5-day-old fetal mice were used as feeder cells. The proliferation of MEFs was arrested by radiation treatment. MEFs meet the criteria for animal-derived raw materials (Public Health Guidelines on Infectious Disease Issues in Xenotransplantation, Ministry of Health, Labor and Welfare, Research and Development Division, 0702001, July 2, 2004). The specifications and test results of the MEF used for the preparation of clinical lots of pHAES-MCB and HLC are presented in Table 2.

Table 4 HLC Characterization and Safety Testing (implemented for each production lot)

Test	Description	Specification prior to freezing of final product
HLC mRNA for AFP and ALB	*Alpha-fetoprotein (AFP) and albumin (ALB) expression levels (compared to the average of all measured spots) by expression analysis using microarray for human gene expression analysis (Agilent, 8x60K).	[AFP+ALB] expression level: more than 50 times the average genotype index
ESC mRNA for LIN28A	*LIN28A expression levels were determined by expression analysis using microarray for human gene expression analysis (Agilent, 8x60K).	LIN28A expression level: less than 3% of undifferentiated pHAES
Cell markers	Immunostaining of Vimentin, Keratin, E-cadherin, α-SMC, TERT, and Lin28.	Confirmation as expected
Ammonia metabolism	Measurement of ammonia concentration by bromocresol green method (Amicheck meter, Arkray, Kyoto, Japan).	Ammonia metabolic capacity of 10 fmol/h/cell or higher
**Test**	**Samples and contents**	**Specifications after final product packaging**
Appearance Inspection	Sample: Final product.<br>Contents: Damage, color, foreign matter check: Exterior appearance inspection.	Shipping containers: No cracks or breaks.<br>Characteristics: Colorless (white) cell suspension in a frozen state.<br>Foreign matter: Not observed.
Total cell count	Sample: Final product from the same lot that was thawed<br>Content: Measured using a hemocytometer after staining with trypan blue.	7.5×10^6 cells/mL or higher
Live cell rate measurement	Sample: Final product from the same lot that was thawed.<br>Content: Measured using a hemocytometer after staining with trypan blue.	70% or more
Sterility	Sample: Final product from the same lot that was thawed.<br>Contents: Automatic measurement by bacillus using a resin bottle.<br>If positive, colonies are detected and identified using an agar medium.	Negative
Mycoplasma	Sample: Final product from the same lot that was thawed.<br>Contents: PCR method.	Negative
Endotoxin	Sample: Supernatant of the same lot of a thawed final product.<br>Contents: Ratio time analysis method by Limulus test.	≦1.0 EU/mL

*For all investigational products, expression using Agilent microarray for gene expression analysis (8x60K) was performed to confirm the validity of the tentative specifications and to obtain data on other appropriate markers for reference in setting specifications after approval.

Quality control of the investigative product

Microbiological safety was performed at external GLP-compliant facilities. Product stability was tentatively set at storage conditions of -135°C and less for 6 months. The effective period after thawing and resuspension of the cells was set at 8 h after thawing (6 h after preparation) at 2-6°C, based on the results of a study at an in-house facility. According to subsequent stability studies, the recommended post-manufacturing storage method and shelf life for the product are as follows: storage temperature should be maintained at -135°C or lower, with a maximum shelf life of 21 months. The cytogenetic stability, infectious

pathogens, karyotyping, and differentiation of pHAES (the raw material of the product) as MCB were stable and safe up to the passage used for production. Animal-derived materials (mice, cattle, and pigs) are used in the manufacturing process, all of which were confirmed to be in compliance with the "Standard for Biological Ingredients" (Ministry of Health, Labor and Welfare Notification No. 37, enacted on February 28, 2008). For the final investigative product, we performed aseptic and mycoplasma tests to confirm the absence of these contaminants. An endotoxin test was performed to confirm that the investigative product met the criteria for an injectable formulation (Table 4).

Tumorigenicity study in the subcutaneous tissue of female nude mice

Tumorigenecity study was performed at an external GLP-compliant facility. A single injection of 1 × 10^7 cells was performed on female nude mice and their tumorigenic potential was examined at 16 weeks. Subcutaneous nodules were analyzed and histopathologically examined at 16 weeks after injection; HeLa S3 cells were used for a control group and the number of cases per group was 10. The examination included general condition, weight measurement, food intake measurement, nodule weight measurement, and histopathological examination at injection site nodules, bilateral lymph nodes, heart, lung, cerebrum, cerebellum, medulla oblongata, spleen, liver, and kidney. Tumorigenicity was evaluated as positive if a tumor was formed at the injection site or metastatic site in one or more cases in the HLC group and if it was determined that the tumor was derived from the injected cells. Histopathologic examination showed granulation tissue at the injection site in one case, but no tumor formation. No tumors were formed in the other cases, suggesting that HLCs are not tumorigenic. In the HeLa S3 group, tumors were formed in all cases.

Biodistribution

Biodistribution study was performed at an external GLP-compliant facility. For ESC-derived HLCs to be effective and safe, they must remain in the liver. To meet the objective of the pharmacokinetic study, we investigated the distribution of ESC-derived HLCs by using PCR to detect Alu gene sequences specific to human DNA [6]. The distribution analysis revealed that there are no human cells in rat organs other than the liver.

Karyotypic analysis

Karyotypic analysis was performed at Nihon Gene Research Laboratories Inc. (Sendai, Japan). Metaphase spreads were prepared from cells treated with 100 ng/mL of Colcemid (Karyo Max, Gibco Co. BRL) for 6 h. The cells were fixed with methanol: glacial acetic acid (2:5) three times and dropped onto glass slides (Nihon Gene Research Laboratories Inc.). Chromosome spreads were Giemsa banded and photographed. A minimum of 10 metaphase spreads were analyzed for each sample and karyotyped using a chromosome imaging analyzer system (Applied Spectral Imaging, Carlsbad, CA).

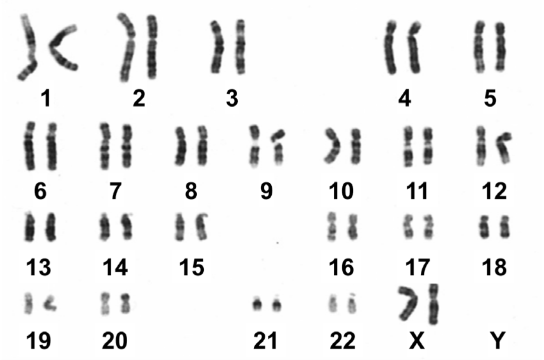

Figure 1. Chromosome analysis of pHAES-WCB cells showed normal karyotypes.

Quantitative RT-PCR

RNA was extracted from cells using the ISOGEN (NIPPON GENE). An aliquot of total RNA was reverse transcribed using an oligo (dT) primer (SuperScript III First-Strand Synthesis System, Invitrogen). For the thermal cycle reactions, the cDNA template was amplified (QuantStudio 12K Flex Real-Time PCR System) with gene-specific primer sets (Supplemental Material, Table 6) using the Platinum Quantitative PCR SuperMix-UDG with ROX (11743-100, Invitrogen) under the following reaction conditions: 40 cycles of PCR (95°C for 15 s and 60°C for 1 min) after an initial denaturation (50°C for 2 min and 95°C for 2 min). Fluorescence was monitored during every PCR cycle at the annealing step. The authenticity and size of the PCR products were confirmed using a melting curve analysis (using software provided by Applied Biosystems) and gel analysis. mRNA levels were normalized using ubiquitin or GAPDH as a housekeeping gene.

Gene chip analysis

High-quality total RNA was isolated from each cell using ISOGEN (NIPPON GENE) following the manufacturer's instructions (Invitrogen, USA). Genomic DNA was eliminated by treatment with DNAse I for 20 min at RT using DNAse IH (Invitrogen, USA). RNA concentration was measured using a Nanodrop ND-1000 spectrophotometer (NanoDrop Technologies, Wilmington, Delaware USA). The purity and integrity of total RNA were determined by 260/280 nm ratio and checked by electrophoresis in Bioanalyzer RNA6000 Nano. About 100 ng of total RNA was used to produce Cyanine 3-CTP labeled cRNA using the Low Input Quick Amp Labeling Kit, One-Color (Agilent Technologies) according to the manufacturer's instructions. Following 'One-Color Microarray Based Gene Expression Analysis' protocol version 6.0 (Agilent Technologies), 2 μg of labeled cRNA was hybridized with a human gene expression microarray 60 K (Agilent Technologies, Santa Clara CA, USA). The microarray workflow quality control was implemented using the Agilent Spike-In Kit which consisted of a set of 10 positive control transcripts optimized to anneal to complementary probes on the microarray with minimal self-hybridization or cross-hybridization. The concentrated Agilent One-Color RNA Spike-In mix stock was diluted in the buffer provided by the kit and mixed with the RNA samples prior to the amplification and labeling process to achieve the relative amounts recommended by the manufacturer. For hybridization, Agilent gene expression microarray 60 K slide (Design ID: 72,363. SurePrint G3 Human Gene Expression 8 × 60 K Microarray Kit, Agilent Technologies) was used. Slides were scanned in an Agilent C Scanner according to the manufacturer's protocol. Signal data were collected with dedicated Agilent Feature Extraction Software (v 11.5.1). Agilent Processed Signals were processed using GeneSpring software (Agilent Technologies).

Measurement of ammonia concentration

Ammonia metabolic capacity assay was performed as previously reported [6]. HLCs (2.5 x10^5) were cultured in 0.2 mL of ammonia-load culture medium at a concentration of approximately 3,000 μg/dl using a 35-mm culture dish. After 20 min preculture at 37°C in a CO_2 incubator, 20 μl of the medium was diluted 10 times with saline, and determined for ammonia concentration with the bromocresol green method. A time course for the ammonia concentration was plotted and the ammonia removal activity was calculated from the slope of an approximate line.

Immunostaining of cells

Thin sections were immunostained with primary antibody reactions using anti-human E-cadherin (BD Transduction Laboratories), anti-human alpha-fetoprotein (AFP) (Dako) and cytokeratins (AE1/3) (712811, NICHIREI) followed by reactions with horseradish peroxidase (HRP)-conjugated secondary antibody (Histofine Simple Stain, Nichirei Bioscience Inc, Tokyo, Japan). Staining was detected by diaminobenzidine and H_2O_2 (Histofine Simple Stain).

Transmission electron microscopy

The samples were fixed using 2.5% glutaraldehyde in 0.1M PBS (pH 7.4) for 2 h. After washing with PBS, samples were postfixed with 2% osmium tetraoxide for 1 h. Samples were dehydrated in a series of ascending ethanol concentrations

and placed in propylene oxide prior to embedding in epoxy resin (Sakura Finetek Japan, Tokyo, Japan). After resin polymerization, sections of approximately 60~80 nm were cut using Ultracuts (Reichert Scientific Instruments Co., Buffalo, NY) and double-stained with uranyl acetate and lead citrate. Electron micrographs were taken using a Hitachi H-7500 transmission electron microscope (Hitachi High-Technologies Corp., Tokyo, Japan).

Array comparative genomic hybridization (CGH)

Genomic mutations may arise during differentiation, posing an additional risk for clinical use. Comparative genomic hybridization (CGH) was used to detect chromosomal and sub-chromosomal aberrations. To analyze genomic structural variants, we chose Agilent SurePrint G3 Human CGH Microarray 8×60K array technology for CGH analysis. Test and reference genomic DNAs (250 ng per sample) were fluorescently labeled with Cy5 (test: SEES2 (pHAES-MCB)) and Cy3 (reference: HLCs) with a Genomic DNA Enzymatic Labeling Kit (Agilent Technologies). All array hybridizations were performed according to the manufacturer's methods. All regions of statistically significant copy number change were determined using Aberration Detection Method-2 (ADM2) algorithms [7]. The ADM2 algorithms identify genomic regions with copy-number differences between the test and the reference based on log2 ratios of fluorescent signals from probes in the interval.

Genomic alteration during the hepatic differentiation

We performed the CGH method with the dye-swap method to investigate whether HLCs had structural alteration (Figure 1). We induced hepatic differentiation of SEES2 cells by embryoid body (EB) formation. The CGH analysis revealed no chromosomal aberrations including gain and loss during the hepatic differentiation.

Reference	Test	ADM2	ADM2
		Threshold=6	Threshold=10
SEES2 (pHAES-MCB)	HLCs	0	0
HLCs	SEES2 (pHAES-MCB)	0	0

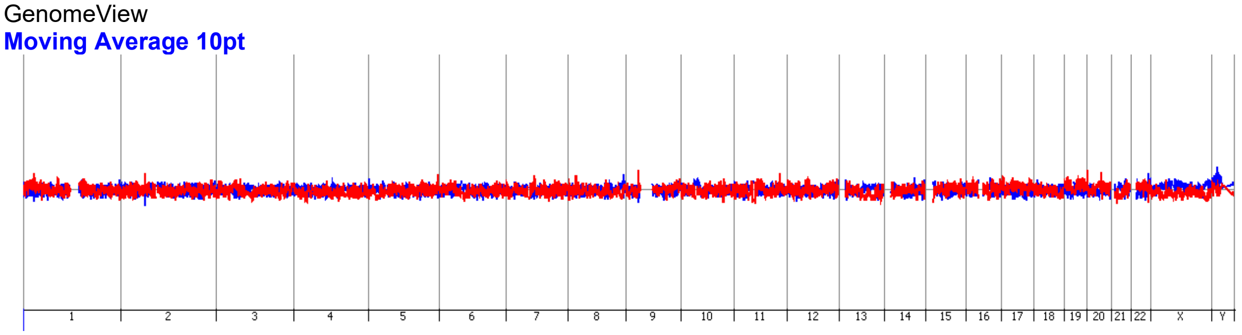

Figure 2. Comparative genomic hybridization (CGH) between SEES-2 cells (pHAES-MCB) and HLCs. The CGH analysis revealed no chromosomal aberrations including gain and loss during the hepatic differentiation.

Table 5 Inclusion/Exclusion Criteria

INCLUSION CRITERIA	• A case of neonatal-onset congenital urea cycle abnormality diagnosed<br>• Cases in which liver transplant surgery is not immediately feasible due to low body weight (less than 6 kg)<br>• Cases in which liver transplantation is considered to be performed when it is determined that liver transplantation can be performed safely<br>• Cases with at least one potential living donor for liver transplantation in the third degree of kinship<br>• Cases in which written consent to participate in this clinical trial has been obtained from the patient's (minor's) surrogate (parents, guardian, etc.)<br>• Patients with cardiopulmonary function as follows:<br>Left ventricular ejection fraction ≥ 50%<br>Percutaneous arterial blood oxygen saturation (SPO2) ≥ 95% at rest
EXCLUSION CRITERIA	• Cases in which HBV, HCV, or HIV infection is suspected from the results of viral tests<br>• Cases with suspected hypersensitivity to ingredients of bovine, porcine, and mouse origin<br>• Cases with serious complications unrelated to the target disease (cardiac disease, pulmonary disease, neurological disease, malignancy, etc.)<br>• Cases in which the surrogate (parents, guardian, etc.) does not give consent for liver transplantation at a time when it is deemed safe to perform liver transplantation<br>• Other cases deemed inappropriate by the investigator (subinvestigator)

Rationale for setting inclusion criteria

(1) Patients whose planned indication is hyperammonemia due to congenital urea cycle abnormalities and of whom the most novel treatment is required

(2) Liver transplantation surgery has poor outcomes in patients weighing less than 6 kg

(3) Because HAES transplantation is intended as a bridge treatment to liver transplantation

(4) Because HAES transplantation is intended as a bridge treatment to liver transplantation

(5) Established in the spirit of GCP and the Declaration of Helsinki

(6) Set for the safety of the subjects.

Rationale for setting exclusion criteria

(1) Set for the safety of the subjects.

(2) The ingredients derived from bovine, porcine, and mouse are used as raw materials or materials for this experimental product, and therefore, the safety of the subjects is taken into consideration.

(3) Set for the safety of the subjects.

(4) Because HAES infusion is intended as a bridge treatment to liver transplantation

(5) Set for the safety of the subjects.

Case 1: citrullinemia type 1, boy

At 2 days old, the patient had seizures, and blood ammonia was 2,026 µg/dl. Continuous hemodiafiltration (CHDF) was started due to blood ammonia level of 2,026 µg/dl. The patient was diagnosed with citrullinemia type 1 based on high levels of urinary orotic acid, blood uracil, and blood citrulline. The diagnosis was confirmed by genetic mutation at 6 days old. The umbilical vein was selected as the route of cell administration. Since angiography showed an open ductus venosus, the ductus venosus was embolized with an AMPLATZER Vascular Plug (Abott Medical, Chicago, IL) and an 18G double-lumen catheter was placed to perform the infusion. The number of cells administered was 1.985 × 10^8 cells (4.0 kg body weight). The protein intake was gradually increased from 0.9 g/kg/day at the time of HLC infusion to 1.5 g/kg/day at 22 days old and to 2 g/kg/day at 43 days old. The ammonia level remained below 150 µg/dl.

Four months after the HLC infusion, he reached a weight of 7 kg. At 5 months old, he received a blood type incompatible living donor liver transplant (A+ to O+) with his father as the donor. The liver graft weight was 220 g and the graft weight/patient weight ratio (GRWR) was 2.85%. Enteral nutrition with GFO (Otsuka pharmaceutical factory, Inc.) was started on postoperative day (POD) 5, and milk was continuously administered via an enteral feeding tube from the next day. Seven days after liver transplantation, liver function deteriorated and ascites increased. A liver biopsy revealed cellular rejection. Steroid bolus injection therapy was performed for POD 5, but ascites did not decrease. A liver biopsy showed residual rejection, and the patient was treated with thymoglobulin. No protein restriction was set, and the type of milk was changed to medium chain triglycerides (MCT) formula on POD 14 because of chylothorax. On POD 21, the patient was transferred from ICU to the general ward. Three months after discharge, there was no recurrence of rejection thereafter.

In the first postoperative year, the developmental evaluation showed postural motor (PM) 86/ cognitive adaptation (CA) 75/ language social (LS) 63/ full (Full) 75, with no obvious seizure waves on EEG and no abnormal signal areas in the cranium on MRI. In the second postoperative year, the developmental evaluation showed a decline in postural motor (PM) 70/ cognitive adaptation (CA) 66/ language social (LS) 59/ full (Full) 65, with no obvious seizure waves on EEG and no abnormal signal areas in the cranium on MRI. There was no decrease in physical development, i.e. -0.2 SD for height and +0.1 SD for weight. Currently, 2 years and 5 months after living donor liver transplantation, he is under outpatient follow-up with immunosuppressive drugs, including Prograf, with no major problems.

Case 2: carbamoyl phosphate synthetase 1 (CPS1) deficiency, boy

The boy had been diagnosed prenatally by chorionic villus sampling with the same genetic mutation as his elder sibling, who had CPS1 deficiency. After birth, genetic testing was also performed on his own blood, and a diagnosis of CPS1 deficiency was confirmed. Hyperammonemia (432 µg/dl) was observed at 2 days old. The umbilical vein was selected as the route of cell administration. After embolization of the vein by AMPLATZER Vascular Plug (Abott Medical, Chicago, IL), an 18G double-lumen catheter was placed and an HLC infusion was performed at 2 and 4 days old. The number of cells administered was 1.495 × 10^8 cells (3.0 kg body weight). The protein intake was gradually increased from 0.25 g/kg/day at the time of HLC infusion to 1.5 g/kg/day at 22 days old. Vomiting and hyperammonemia were observed 22 days after the infusion. The patient was diagnosed with hypertrophic pyloric stenosis by ultrasound. Atropine sulfate therapy was not effective, and Ramstedt surgery was performed 48 days after the infusion. Ammonia blood level temporarily increased to 417 µg/dl. CHDF was started, but withdrawn within 1 day.

However, he developed hyperammonemia (525 µg/dl) due to an upper respiratory tract infection by enterovirus/rhinovirus. A blood group-matched brain-dead liver transplantation (B+ to AB+) was performed, with a liver graft weight of 239 g and a GRWR of 3.27%. Enteral nutrition with GFO was started from POD 4, and milk was continuously administered via an enteral feeding tube from day 6. Total bilirubin was elevated on POD 9, and liver biopsy showed moderate to severe cellular rejection. The patient was treated with steroid pulse therapy for 4 days. A follow-up liver biopsy showed residual rejection, and the patient was treated with thymoglobulin. The patient was transferred from ICU to the general ward on POD 22. Three months after discharge, the bile duct tube was removed, and there was no recurrence of rejection or hyperammonemia. At the 1-year postoperative developmental evaluation, his intellectual level was maintained with postural motor (PM) 126/ cognitive adaptation (CA) 91/ language social (LS) 107/ full (Full) 99. Currently, 1 year and 9 months have passed since the living donor liver transplantation, and he is under outpatient observation with immunosuppressive drugs such as Prograf, but there are no major problems. There is no decrease in physical development, i.e. height -0.7 SD and weight +1.0 SD.

Case 3: ornithine carbamylase (OTC) deficiency, boy

The patient was intubated at 5 days old with frequent apneic attacks. Hyperammonemia (2,178 μg/dl) was observed. The pupils were dilated and the whole brainstem reflexes were absent. EEG showed severe hypoactivity, but ammonia level decreased to 128 μg/dl, and neurological findings became normalized after initiation of CHDF and drug therapy. The patient was diagnosed with OTCD by genetic testing at 12 days old. HLCs were infused at 45 and 47 days old. The umbilical vein was selected as the route, and an 18G double-lumen catheter was placed without embolization of the venous duct, and 1.6 × 10^8 cells (3.2 kg body weight) in total were infused. The protein intake was gradually increased from 1.0 g/kg/day at the time of HLC infusion to 2.0 g/kg/day at 48 days old. Ammonia levels were maintained at 40 μg/dl; by the end of 4 months, ammonia levels had increased to 121 μg/dL.

Five months after the HLC infusion, a blood type-matched brain-dead liver transplant (O+ to B+) was performed at a weight of 7.2 kg with a graft weight of 290 g and a GRR of 3.97%. Enteral feeding with GFO was started from POD 4, and milk via enteral feeding tube was continued from POD 6. Hepatic function decreased (elevated AST, ALT, and LDH) on POD 10, and a liver biopsy revealed moderate to severe cellular rejection. Steroid pulse therapy was performed for 2 days. Liver function decreased further and the patient was treated with thymoglobulin. The patient was transferred from ICU to the general ward on POD 18; he was discharged on POD 53 and the tube was removed 3 months later. Thereafter, there was no rejection and no hyperammonemia.

In the first postoperative year, an MRI examination revealed abnormal signal areas in both the left and right corona radiata; the abnormal signals did not progress over time. Additionally, no obvious seizure waves were detected on EEG. At 1 and 5 months postoperatively, the developmental assessment showed that the intellectual level was maintained, with postural motor (PM) 75/ cognitive adaptation (CA) 82/ language social (LS) 90/ full (Full) 83. There was a slight decrease in physical development, -1.0SD for height and -0.6SD for weight. He is under outpatient follow-up with immunosuppressive drugs, including Prograf and CELLCEPT, with no major problems.

Case 4: OTC deficiency, boy

The patient exhibited decreased response, muscle tone, and irregular breathing at 3 days old, with hyperammonemia (2,100 µg/dl). When the patient was transported to our hospital, ammonia level had risen to 2,679 µg/dl. At 9 days old, when he was diagnosed with OTCD by genetic testing, his ammonia level was stable at 40-60 µg/dl by CHDF and drug therapy. The patient was weaned from CHDF and weighed 3.6 kg. The patient was placed with an 18G double-membrane catheter, and he underwent HLC infusion at 16 and 18 days old while monitoring portal vein pressure and portal blood flow. The total number of cells administered was 1.7 × 10^8 cells (3.4 kg body weight). He was diagnosed with hypertrophic pyloric stenosis by ultrasound 11 days after the infusion, and atropine was administered.

Four months after the HLC infusion, he reached a weight of 6 kg and underwent a blood type-matched living donor liver transplant (A+ to A+) with his father as the donor. The transplanted liver was in the lateral graft area, with a graft weight of 151 g and a GRWR of 2.51%. Enteral nutrition using GFO was started on POD 5, and milk was continuously administered through an enteral feeding tube on POD 5. On POD 7, the patient was transferred from the ICU to the general ward. Because of increasing ascites, a liver biopsy was performed on POD 13 in consideration of possible rejection. A liver biopsy showed no evidence of rejection, but the patient had a severe fatty liver. Protein intake was not restricted and ammonia was not elevated. The patient was discharged on POD 57. Three months after discharge, the bile duct tube was removed, and there was no recurrence of rejection or hyperammonemia. Six months after the transplantation, the patient was still short and underweight, with a height of -1.4SD and a weight of -0.8SD, although physical development was observed. No neurologically developmental delays were apparent; outpatient follow-up is ongoing.

Case 5: CPS1 deficiency, girl

The patient was on a ventilator due to decreased feeding and hypercapnia with hyperammonemia (1,578 µg/dl) at 3 days old. Amino acid analysis showed high levels of glutamine, glutamic acid, and alanine, and low levels of citrulline, suggesting CPS1D. She had 4 episodes of recurrent hyperammonemia after withdrawal from dialysis, and brain damage was increasing. CPS1D was confirmed by genetic testing. The patient underwent HLC infusion at 58 and 61 days old while monitoring portal vein pressure and portal blood flow. The umbilical vein was selected as the route, and an 18G double-lumen catheter was placed without embolization of the venous duct. The total number of cells administered was 1.7 × 10^8 cells (3.3 kg body weight). Protein intake was 1.0 g/kg/day at the time of HLC transplantation, and the protein dose was gradually increased after the HLC infusion. The protein intake was 1.5 g/kg/day at 70 days old. MRI showed atrophy of the cerebrum, decreased white matter volume, and enlarged ventricles, and spiny waves of suspected epileptogenicity were observed on EEG.

The patient reached a weight of 6 kg at 3 months after the HLC infusion and underwent blood group-matched living donor liver transplantation (O+ to A+) with her mother as the donor. The transplanted liver was in the lateral graft area with a graft weight of 254 g and a GRWR of 4.1%. The patient was transferred from the ICU to the general ward on POD 9. Her postoperative liver function was stable, and there was no evidence of rejection. Physical development showed height -2.7SD and weight -1.6SD, and both height and weight began falling behind the growth curve. Hepatic function is stable with Prograf. Outpatient follow-up is ongoing with anticonvulsant medication.

Table 6 Primers for qRT-PCR

Gene product	Forward primers (5'–3')	Reverse primers (5'–3')
Ub	GGAGCCGAGTGACACCATTG	CAGGGTACGACCATCTTCCAG
AFP	AGCTTGGTGGTGGATGAAAC	CCCTCTTCAGCAAAGCAGAC
ALB	TGGCACAATGAAGTGGGTAA	CTGAGCAAAGGCAATCAACA
OTC	ACCTTCAGGCAGCTACTCCA	GCCGCTTTTTCTTCTCCTCT
ARG1	TGATGTTGACGGACTGGACC	TGCAACTGCTGTGTTCACTG

References

1. Akutsu H, Nasu M, Morinaga S, Motoyama T, Homma N, Machida M, et al. In vivo maturation of human embryonic stem cell-derived teratoma over time. Regen Ther. 2016;5:31–9.

2. Akutsu H, Machida M, Kanzaki S, Sugawara T, Ohkura T, Nakamura N, et al. Xenogeneic-free defined conditions for derivation and expansion of human embryonic stem cells with mesenchymal stem cells. Regen Ther. 2015;1:18–29.

3. Umezawa A, Sato Y, Kusakawa S, Amagase R, Akutsu H, Nakamura K, et al. Research and Development Strategy for Future Embryonic Stem Cell-Based Therapy in Japan. JMA J. 2020;3:287–94.

4. Tate JG, Bamford S, Jubb HC, Sondka Z, Beare DM, Bindal N, et al. COSMIC: the Catalogue Of Somatic Mutations In Cancer. Nucleic Acids Res. 2019;47:D941–7.

5. Shibata list [Internet]. [cited 2023 Feb 9]. Available from: http://togodb.org/db/shibata_list

6. Enosawa S. Isolation of GMP Grade Human Hepatocytes from Remnant Liver Tissue of Living Donor Liver Transplantation. In: Stock P, Christ B, editors. Hepatocyte Transplantation: Methods and Protocols. New York, NY: Springer New York; 2017. p. 231–45.

7. Lipson D, Aumann Y, Ben-Dor A, Linial N, Yakhini Z. Efficient calculation of interval scores for DNA copy number data analysis. J Comput Biol. 2006;13:215–28.
